# Assessing the Correlation Between Langerhans Cells Population and Prognosis of Tongue Squamous Cell Carcinoma

**DOI:** 10.1002/cre2.70080

**Published:** 2025-01-30

**Authors:** Narges Ghazi, Nasrollah Saghravanian, Pooya Saeedi, Mohammad Mahdi Maboudinezhad

**Affiliations:** ^1^ Department of Oral and Maxillofacial Pathology, School of Dentistry Mashhad University of Medical Sciences Mashhad Iran; ^2^ School of Dentistry Mashhad University of Medical Sciences Mashhad Iran

**Keywords:** CD1a, Langerhans cell, prognosis, tongue squamous cell carcinoma

## Abstract

**Background and Objective:**

Tongue squamous cell carcinoma (TSCC) is the most prevalent oral cancer. Despite considerable advancements in treatment, the 5‐year survival rate remains relatively unchanged. Langerhans cells (LCs) play an important role in antitumor immunity. Therefore, we attempt to evaluate the correlation between the LC count and disease prognosis.

**Materials and Methods:**

Histopathologic slides from 24 cases, with at least 2 years of follow‐up, were selected and categorized into early‐stage (12 cases) and advanced‐stage (12 cases) groups. An additional 12 slides of normal tissue comprised the control group. Immunohistochemical staining with the CD1a marker was performed to analyze the density of LCs. Statistical analysis assessed the impact of CD1a immune expression on patient survival and other variables such as age, gender, stage, and histopathological grade.

**Results:**

Comparison of CD1a+ cell counts across the three groups revealed a significant decrease in the advanced group. Furthermore, a lower count of CD1a+ cells correlated with poorer disease‐free survival (DFS) (*p* < 0.001) and overall survival (OS) (*p* = 0.049). Although the CD1a+ cell count did not independently affect OS significantly (*p* = 0.210), it did show a significant impact on DFS as an independent variable (*p* = 0.002).

**Conclusion:**

The significant correlation between CD1a expression and patients' prognosis and survival rates suggests that CD1a+ cells could serve as a crucial prognostic factor in the management and treatment of TSCC.

## 1 Introduction

Oral cavity cancer ranked as the 18th most prevalent malignancy globally in 2020, posing a substantial health challenge (Sung et al. 2021). Squamous cell carcinomas of the oral cavity (OSCC), which constitute over 90% of oral cavity cancers, originate from the squamous epithelium. These cancers show a 2.2 male‐to‐female ratio, with the majority of cases occurring in individuals aged 50–70 years (Vered et al. 2019; Mirhashemi et al. 2023). Annually, more than 400,000 cases of oral cancer are diagnosed, with over half attributed to the tongue (Ganly, Patel, and Shah 2012; Warnakulasuriya 2009).

In contrast to other malignancies, tongue squamous cell carcinoma (TSCC) is on the rise, particularly among individuals aged 20–44 years (Rethman et al. 2010; Vered, Dayan, and Salo 2011). In many countries, tongue cancer is recognized as a significant public health concern due to its elevated morbidity and mortality rates (Annertz et al. 2012; Camisasca et al. 2011; Alho et al. 1999; Wang et al. 2013).

TSCC displays an aggressive nature, leading to significant disruptions in speech, mastication, and swallowing (Jemal et al. 2017). Notably, TSCC has heightened rates of recurrence and metastasis, coupled with diminished survival rates, compared to SCC in other oral and head and neck regions (Jemal et al. 2010). The challenging prognosis of TSCC underscores the complexities associated with invasion and metastasis (Manning and Cantley 2007). Although indications suggest a potential biological distinction for TSCC, this remains a subject of debate requiring extensive investigation (Kalluri and Zeisberg 2006; Rodrigues‐Lisoni et al. 2010).

The primary treatment protocol for TSCC involves surgery and radiotherapy to eliminate malignant epithelial cells. Unfortunately, significant advancements in the treatment of TSCC patients have not taken place over the past four decades with this therapeutic approach, and the overall survival (OS) rate remains low, with a 50% 5‐year survival rate (Hiemer et al. 2015).

An ideal prognostic factor for predicting TSCC recurrence has yet to be established. Some studies highlight the significance of surgical margin status, whereas others do not demonstrate a clear association between this factor and recurrence or survival rates (Yanamoto et al. 2012; Brandwein‐Gensler et al. 2005).

Among the cells playing a crucial role in the immune system's function against malignancies are Langerhans cells (LCs), which present internal or external antigens to T cells. CD1a, a membrane‐bound peptide expressed in LCs, facilitates the presentation of lipid and glycolipid antigens to T cells. Consequently, these cells represent a significant target for therapeutic and preventive processes in cancer (Stoitzner, Sparber, and Tripp 2010; Schuler, Schuler‐Thurner, and Steinman 2003).

In this study, we evaluated the relationship between the number of LCs (assessed through CD1a expression) in patients with TSCC compared to normal tissue. Additionally, we examined the correlation between these cells and patients' prognoses.

## 2 Methods and Materials

### 2.1 Patients

This study included patients diagnosed with TSCC whose medical records and pathological specimens were sourced from the Oncology Department of Omid Hospital and the Oral and Maxillofacial Pathology Department at Mashhad University of Medical Sciences, Iran. Convenience sampling was used to select cases from the pathology archives based on their availability and alignment with the study's inclusion and exclusion criteria. Inclusion criteria comprised patients with comprehensive clinical records, a follow‐up duration exceeding 2 years, and suitable pathological specimens for immunohistochemistry. Exclusion criteria included patients with incomplete medical records, inadequate pathological specimens for analysis, or significant co‐morbidities potentially influencing survival outcomes independently of TSCC progression, such as HPV infection. Relevant information and participant contact details were carefully extracted from the archives, including clinicopathological data such as age, gender, stage, histopathological grade, recurrence, and survival rate.

### 2.2 Specimen Characteristics

Archived formalin‐fixed, paraffin‐embedded tissue blocks were retrieved for analysis. Two 4–5 μm‐thick sections were prepared from each block and validated for quality and quantity by two Oral and Maxillofacial Pathology professors (N.G. and N.S.).

### 2.3 Assay Methods

Immunohistochemical staining was performed using the BOND Ready‐to‐Use Primary Antibody CD1a (MTB1) kit (Leica Biosystems Newcastle Ltd., United Kingdom) following the manufacturer's protocol. Sections were deparaffinized, rehydrated, and subjected to antigen retrieval using the automated Leica Bond‐Max system. Stained slides were subjected to quality control procedures to ensure reproducibility and reliability of results. CD1a‐positive dendritic cells (DCs) were identified and quantified. For each sample, three representative high‐cellularity “hot spot” fields within the invasive tumor component were identified under ×100 magnification. The mean number of CD1a‐positive DCs across these fields was recorded for each sample (Minesaki et al. 2021; Silva et al. 2020). Immunohistochemical evaluations were conducted in a manner blinded to the study endpoints to prevent bias.

### 2.4 Study Design

This biomarker retrospective study was conducted by examining medical records and archived pathological specimens from the Oncology Department of Omid Hospital and the Oral and Maxillofacial Pathology Department at Mashhad University of Medical Sciences, Iran. Cases were selected from a time period spanning from January 2008 to December 2018, with follow‐up data collected through December 2020.

Subsequently, a total of 36 samples were chosen from the pathology archives, divided into three groups: the control group, sourced from mucocele specimens, included epithelial and normal connective tissues without inflammation or with only mild inflammation and no epithelial dysplasia. There were 12 cases at Stage I or II and 12 cases at Stage III or IV based on the TNM classification. The early‐stage TSCC group included samples from Stages I and II TSCCs, whereas the advanced‐stage TSCC group comprised samples from Stages III and IV TSCCs (Mohtasham et al. 2014). Additionally, TSCC samples were classified as low grade and high grade.

Matching criteria such as age and gender distribution were assessed to ensure comparability across groups. Moreover, disease‐free survival (DFS) was calculated from the time of pathological diagnosis to the first recurrence, whereas OS spanned from the time of pathological diagnosis to the last follow‐up or the date of death. The study protocol was approved by the Ethics Committee of Mashhad University of Medical Sciences under the code IR.MUMS.DENTISTRY.REC.1401.150.

Sample size calculation was based on the findings of Upadhyay et al. (2012). With a target power of 80% and a significance level of α = 0.05, the minimum required sample size was determined to be 36 samples, with 12 samples allocated to each group. PASS software (version 2021; NCSS LLC, Kaysville, Utah, USA) was utilized for sample size determination.

### 2.5 Statistical Analysis Methods

All statistical analyses were performed using SPSS software (version 23; SPSS Inc., Chicago, IL, USA). Survival analyses, including Kaplan–Meier and Cox regression analyses, were conducted. The chi‐square test was used for comparison between groups. Statistical significance was established at *p* < 0.05.

## 3 Results

**Figure 1:**
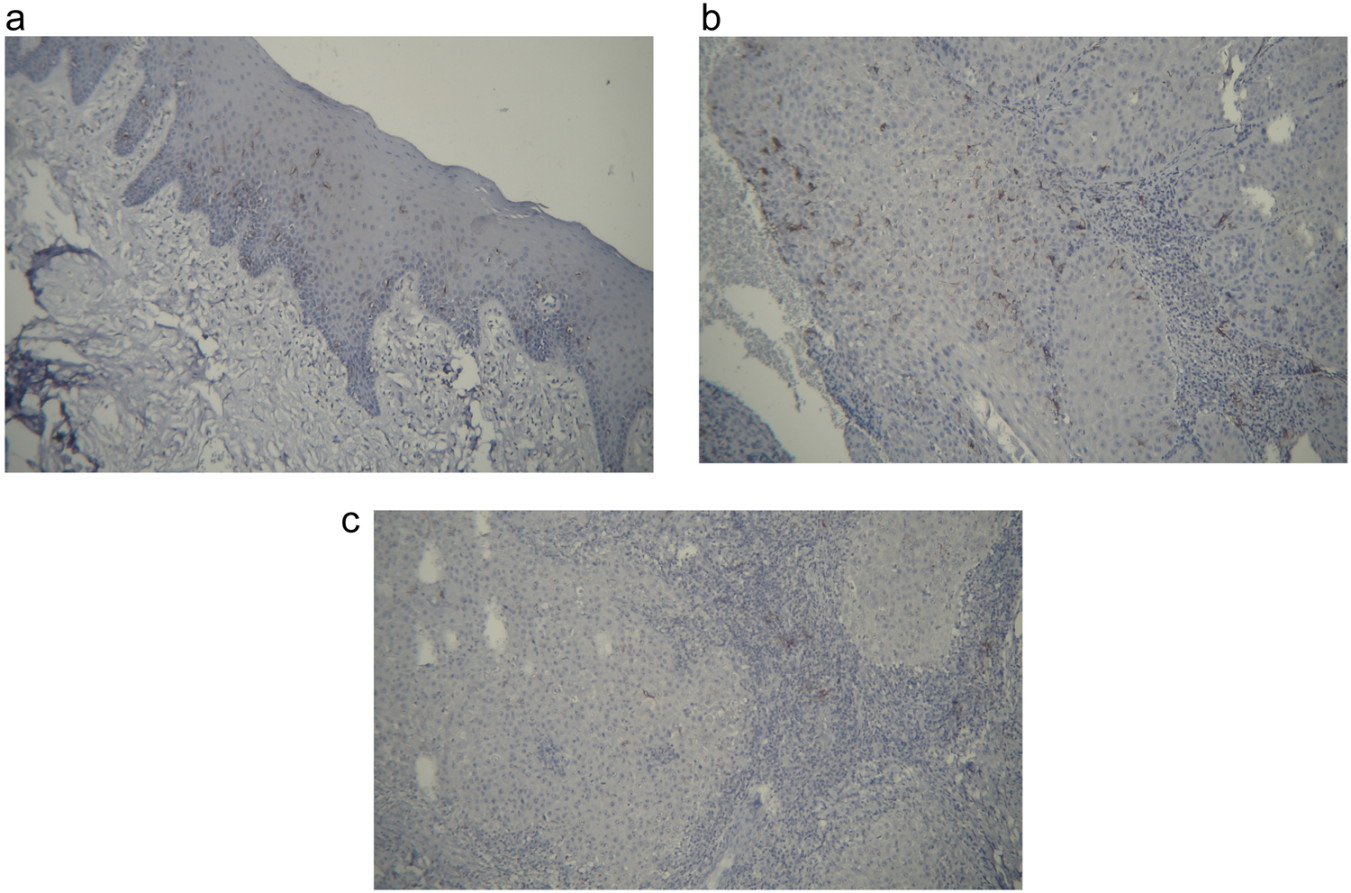
(a) High CD1a expression in Langerhans cells in the normal group (×40). (b) High expression of CD1a in Langerhans cells in the early‐stage group (×100). (c) Low expression of CD1a in Langerhans cells in the advanced‐stage group (×100).

Among the 36 patients, the average age was 60.11 ± 11.73 years, ranging from 32 to 78 years. Table 1 shows an equal distribution of 50% females and 50% males across all three groups, indicating no significant gender difference among the groups (*p* = 1.000). Similarly, no statistically significant difference in age was observed among the groups (*p* = 0.236). Positive immunoreactivity was observed in 100% of the control group, 75% of the early‐stage group, and 41.7% of the advanced‐stage group, with a significant difference (*p* = 0.002). Additionally, the average CD1a gene expression in the control, early‐stage, and advanced‐stage groups was 23.17, 20.11, and 3.20, respectively, indicating a significant difference among the groups (*p* < 0.001) (Figure 1).

**Table 1:** Chi‐square analysis examining differences in gender, age, immunoreactivity, and CD1a expression among three study groups.

Variables		Groups			*p* value
		Control	Early Stage	Advanced Stage	
Gender (*N/%*)	Male	6/50	6/50	6/50	1.000
	Female	6/50	6/50	6/50	
Age, years (M ± SD)		58.08 ± 12.53	57.42 ± 13.13	64.83 ± 8.45	0.236
Immunoreactivity (*N/%*)	Negative	0/0	3/25	7/58.3	0.002*
	Positive	12/100	9/75	5/41.7	
CD1a expression (M ± SD)		23.17 ± 5.55	15.08 ± 11.92	1.33 ± 1.72	0.000*

*Note:* A significant difference between groups is indicated by an asterisk (*) (*p* < 0.05). A 95% confidence interval of the mean was estimated in the analysis.

**Figure 2:**
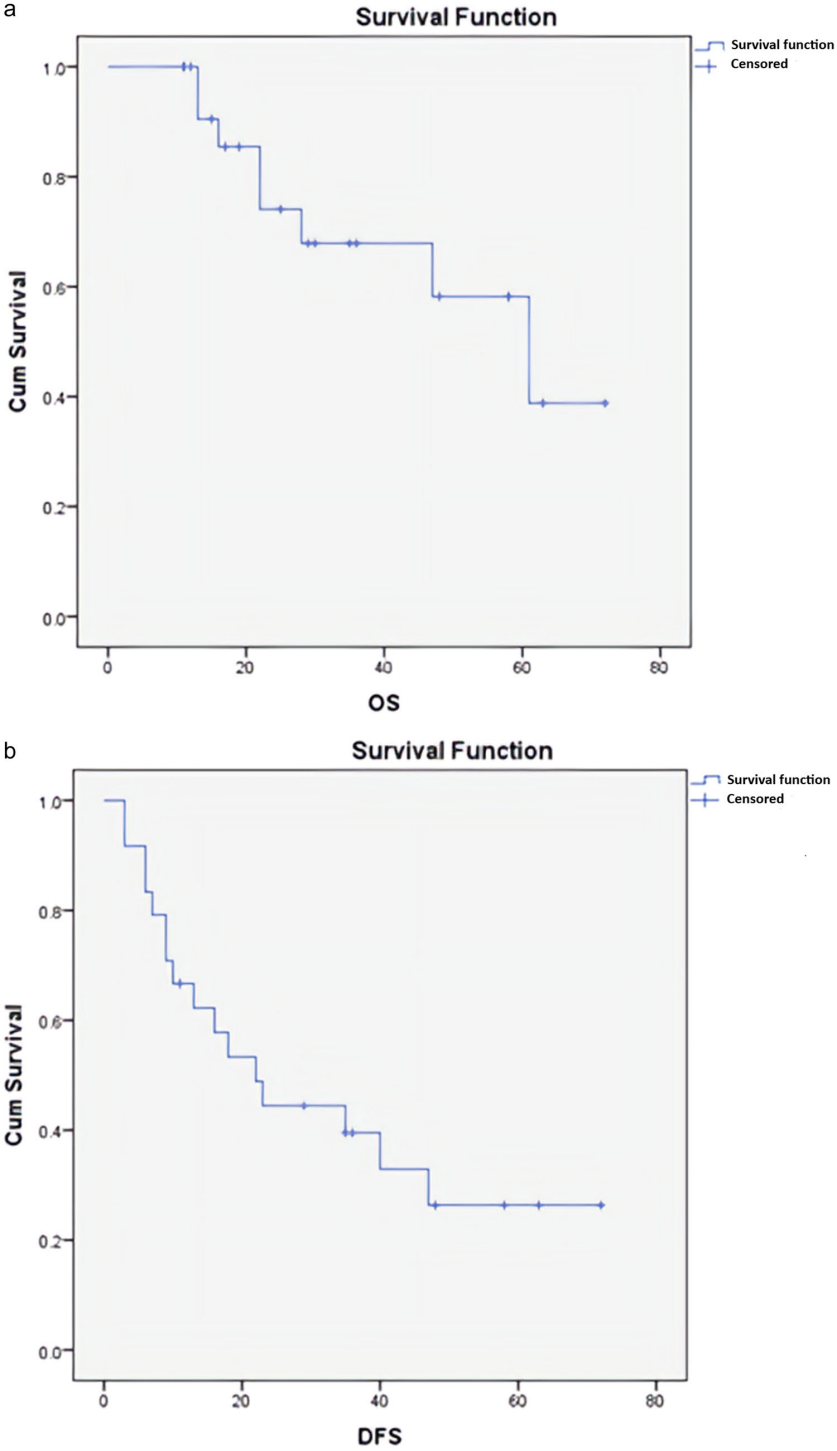
(a) Overall survival (OS). (b) Disease‐free survival (DFS).

No statistically significant association was found between CD1a expression and patient age (*p* = 0.471) or histopathological grade (*p* = 0.457) (Table 2). Kaplan–Meier survival analysis revealed a median OS of 61 months, with a 3‐year survival rate of 67.9% ± 11% and a 5‐year survival rate of 58.2% ± 13%. The median DFS was 22 months, with a 3‐year survival rate of 39.5% ± 10.3% and a 5‐year survival rate of 26.3% ± 10.2% (Figure 2).

**Table 2:** Chi‐square analysis investigating the difference in age and disease grade between two CD1a expression groups.

Variables		CD1a expression		*p* value
		Negative	Positive	
Age, years (M ± SD)		57.8 ± 11.8	61 ± 11.81	0.471
Grade (*N/%*)	Low	5/50	9/64.3	0.457
	High	5/50	5/35.7	

*Note:* A 95% confidence interval of the mean was estimated in the analysis.

**Figure 3:**
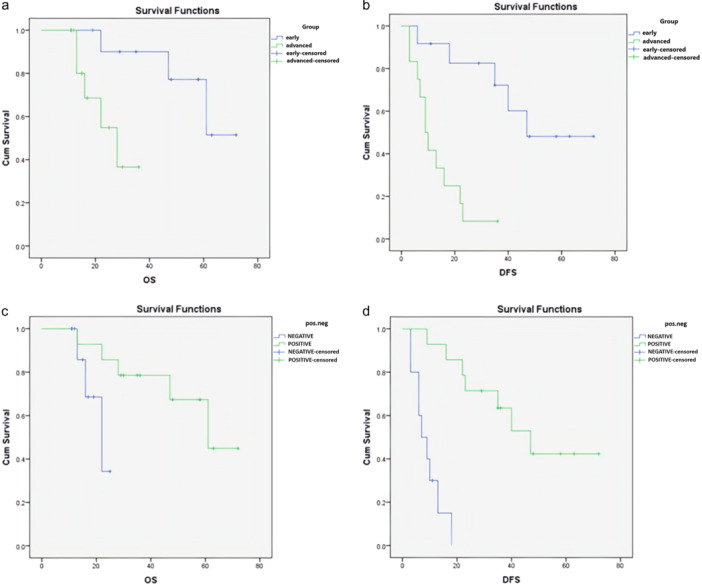
(a) Effect of disease stage on overall survival. (b) Effect of disease stage on disease‐free survival. (c) Effect of CD1a on overall survival. (d) Effect of CD1a on disease‐free survival.

An analysis of the effect of disease stage on OS revealed a 3‐year survival rate of 90% ± 9.5% in early‐stage patients compared to 18.8% ± 36.6% in advanced‐stage patients. The mean OS for early‐ and advanced‐stage patients was 61 and 26 months, respectively, showing a significant impact of disease stage on OS (*p* = 0.017). Regarding DFS, early‐stage patients had a 3‐year survival rate of 72.2% ± 13.8%, whereas advanced‐stage patients had a rate of 8.3% ± 8%. The mean DFS for these groups was 51 and 13 months, respectively, indicating a significant impact of disease stage on DFS (*p* < 0.001) (Figure 3 and Table 3).

**Table 3:** Log‐rank Chi‐square analysis examining the influence of disease stage and CD1a expression on overall survival and disease‐free survival.

Variables			Median survival	Mean survival	Std. error	*p* value
Overall survival	Stage	Early	—	60.96	5.35	0.017*
		Advanced	28	25.73	28	
	CD1a expression	Negative	22	—	4.54	0.049*
		Positive	61	—	12.83	
Disease‐free survival	Stage	Early	47	50.88	7.15	0.000*
		Advanced	9	13.08	2.69	
	CD1a expression	Negative	7	—	2.37	0.000*
		Positive	47	—	8.51	

*Note:* A significant impact is indicated by an asterisk (*) (*p* < 0.05). A 95% confidence interval of the mean was estimated in the analysis.

Furthermore, the median OS in the negative and positive CD1a expression groups was 22 and 61 months, respectively, demonstrating a significant effect of CD1a expression on OS (*p* = 0.049). For DFS, the median survival was 7 months in the negative CD1a group and 49 months in the positive group, indicating a significant correlation between CD1a expression and DFS (*p* < 0.001) (Figure 3 and Table 3).

As both disease stage and CD1a gene expression significantly influenced survival, Cox regression analysis was conducted to evaluate their relationship with survival outcomes. According to Table 4, neither disease stage nor CD1a expression significantly impacted OS (*p* = 0.083 and *p* = 0.210, respectively). However, a significant correlation between both CD1a expression and disease stage with DFS was observed (*p* = 0.006 and *p* = 0.002, respectively).

**Table 4:** Cox regression analysis examining the relationship between disease stage and CD1a expression, controlling for each other, and their impact on overall survival and disease‐free survival.

Variables		B	Standard Deviation	*p* value
Overall survival	Stage	−1.959	1.130	0.083
	CD1a expression	1.215	0.969	0.210
Disease‐free survival	Stage	−2.032	0.739	0.006*
	CD1a expression	2.993	0.968	0.002*

*Note:* A significant impact is indicated by an asterisk (*) (*p* < 0.05). A 95% confidence interval of the mean was estimated in the analysis.

The staining pattern of LCs was observed in both the epithelial and stromal compartments. LC was distributed intraepithelially and within the connective tissues of the tumor microenvironment. For quantification, LCs with positive immunoreactivity were collectively counted in both the epithelial and stromal compartments to provide an overall representation of their presence. Differences in the distribution of LC between the epithelial and stromal compartments were noted, with a higher concentration generally observed in the epithelial regions compared to the stroma.

## 4 Discussion

In this study, we conducted a quantitative analysis of LCs by assessing the expression of the CD1a marker in control, early‐stage, and advanced‐stage groups. Our statistical analyses revealed a significant reduction in CD1a expression in the advanced‐stage group compared to both the control and early‐stage groups. Furthermore, we evaluated the correlation between CD1a expression and clinical outcomes such as DFS and OS. The findings revealed a significant association, indicating that heightened CD1a expression corresponds to improved survival in TSCC patients.

No significant differences were observed between lesion grade and CD1a expression. This outcome was anticipated, as our study's primary focus was not on the relationship between marker expression and histopathological grade. Additionally, the study highlighted a nonsignificant relationship between marker expression and patient age or gender, indicating that these demographic variables had minimal influence on CD1a expression.

Our findings align with the research conducted by Jardim et al. (2018), who explored the density of CD1a+ and CD83+ cells in patients with oral SCC. Their study demonstrated a strong correlation between reduced CD1a+ cell density in tumor‐adjacent tissues and adverse clinical outcomes, including DFS and OS. However, in our study, Cox regression analysis did not establish a significant association between the CD1a+ cell count and OS. This discrepancy could be attributed to differences in sample size and follow‐up duration between the two studies.

Similarly, Gomes et al. (2016) observed a reduction in LC count in well‐differentiated lower lip SCC, attributing this decrease to prolonged UV exposure. Our findings corroborate this observation, emphasizing the pivotal role of the immune system in SCC pathogenesis.

Notably, the study carried out by Maraee et al. (2020) focused on tumor‐infiltrating LCs in non‐melanotic skin cancers. Their results showed fewer LCs in larger tumors and in SCC compared to BCC, consistent with our findings and underscoring the critical role of LCs in tumor progression.

Contrasting results were reported by Minesaki et al. (2021), who investigated the role of CD1a+, S100+, and CD8+ cells in advanced oropharyngeal cancer. They observed lower DSS and OS in groups with elevated LC counts. The authors suggested that these contradictions might stem from the diverse functions of LCs across different body organs. This highlights the need for further studies with larger sample sizes to provide more robust and conclusive evidence regarding the role of LCs in cancer prognosis.

CD1a, as a promising biomarker, has the potential to significantly impact the assessment of malignancy prognosis when used alongside established staging systems. This suggests that decisions regarding the extent of invasiveness during lesion excision, crucial for effective surgical interventions, can be informed by the presence of CD1a. Early evaluation of this biomarker could improve treatment strategies, reduce disease recurrence, and enhance overall patient survival.

In cancer research, numerous studies have explored the therapeutic or suppressive potential of DCs (Stoitzner, Sparber, and Tripp 2010; Schuler, Schuler‐Thurner, and Steinman 2003). Based on the findings of these studies and the present research, utilization of these cells could lead to remarkable advancements in cancer treatment and prevention.

Future studies should evaluate additional pathological parameters such as the degree of differentiation, tumor budding, and pattern of invasion, which are assessable on H&E stainings and have proven prognostic value. Investigation of correlations between these features and LC distribution may provide deeper insights into the tumor microenvironment and its impact on survival outcomes.

## 5 Conclusion

The significant association between decreased CD1a expression and reduced patient survival, as well as disease progression, highlights the potential of CD1a as an independent prognostic factor in the assessment of TSCC patients. Furthermore, considering the critical role of LCs in presenting tumor antigens and the adverse effects of their depletion on cancer cell invasion and malignancy, these cells present themselves as promising candidates for therapeutic interventions aimed at improving outcomes for affected patients.

## Author Contributions

**Narges Ghazi:** conceptualization, methodology, resources, writing – review and editing, and project administration. **Nasrollah Saghravanian:** conceptualization, methodology, validation, supervision, and project administration. **Pooya Saeedi:** investigation, visualization, and writing – original draft and writing – review and editing. **Mohammad Mahdi Maboudinezhad:** conceptualization, validation, investigation, writing – review and editing, and visualization.

## Conflicts of Interest

The authors declare no conflict of interest.

## Data Availability

The data that support the findings of this study are available from the corresponding author upon reasonable request.
